# High-flow nasal oxygen *versus* conventional oxygen therapy and noninvasive ventilation in COVID-19 respiratory failure: a systematic review and network meta-analysis of randomised controlled trials

**DOI:** 10.1016/j.bja.2023.12.022

**Published:** 2024-02-02

**Authors:** Walter Pisciotta, Alberto Passannante, Pietro Arina, Khalid Alotaibi, Gareth Ambler, Nishkantha Arulkumaran

**Affiliations:** 1 Bloomsbury Institute of Intensive Care Medicine, Division of Medicine, University College London, London, UK; 2 Department of Statistical Science, University College London, London, UK

**Keywords:** acute respiratory distress syndrome (ARDS), COVID-19, high-flow nasal oxygen, noninvasive ventilation, respiratory failure, ventilation

## Abstract

**Background:**

Noninvasive methods of respiratory support, including noninvasive ventilation (NIV), continuous positive airway pressure (CPAP), and high-flow nasal oxygen (HFNO), are potential strategies to prevent progression to requirement for invasive mechanical ventilation in acute hypoxaemic respiratory failure. The COVID-19 pandemic provided an opportunity to understand the utility of noninvasive respiratory support among a homogeneous cohort of patients with contemporary management of acute respiratory distress syndrome. We performed a network meta-analysis of studies evaluating the efficacy of NIV (including CPAP) and HFNO, compared with conventional oxygen therapy (COT), in patients with COVID-19.

**Methods:**

PubMed, Embase, and the Cochrane library were searched in May 2023. Standard random-effects meta-analysis was used first to estimate all direct pairwise associations and the results from all studies were combined using frequentist network meta-analysis. Primary outcome was treatment failure, defined as discontinuation of HFNO, NIV, or COT despite progressive disease. Secondary outcome was mortality.

**Results:**

We included data from eight RCTs with 2302 patients, (756 [33%] assigned to COT, 371 [16%] to NIV, and 1175 [51%] to HFNO). The odds of treatment failure were similar for NIV (*P*=0.33) and HFNO (*P*=0.25), and both were similar to that for COT (reference category). The odds of mortality were similar for all three treatments (odds ratio for NIV *vs* COT: 1.06 [0.46–2.44] and HFNO *vs* COT: 0.97 [0.57–1.65]).

**Conclusions:**

Noninvasive ventilation, high-flow nasal oxygen, and conventional oxygen therapy are comparable with regards to treatment failure and mortality in COVID-19-associated acute respiratory failure.

**Prospero registration:**

CRD42023426495.

**Editor's key points**

• In adult patients with non-COVID-19 acute hypoxaemic respiratory failure, use of noninvasive ventilation (NIV) or high-flow nasal oxygen (HFNO) is associated with a reduction in the rates of intubation and mortality compared with conventional oxygen therapy (COT).
• In this systemic review with meta-analysis, of a homogeneous cohort of patients with acute respiratory failure treatment failure and mortality rates in patients with COVID-19 were comparable between NIV, HFNO, and COT.
• Additional trials with present-day management of acute respiratory distress syndrome are required to evaluate the efficacy of NIV, HFNO, and COT within homogeneous cohorts of patients.

Invasive mechanical ventilation (IMV) is used to support patients with severe acute respiratory failure (ARF). However, IMV requires the use of additional treatment (including sedation), requires significantly more resources, and is associated with potential inadvertent harm to patients (ventilator-associated pneumonia and ventilator-induced lung injury). Noninvasive methods of respiratory support, including noninvasive ventilation (NIV), continuous positive airway pressure (CPAP), and high-flow nasal oxygen (HFNO), are potential strategies to prevent progression to requirement for IMV in acute hypoxaemic respiratory failure.

In adult patients with non-COVID-19 acute hypoxaemic respiratory failure, the use of noninvasive respiratory support is associated with a reduction in the rates of tracheal intubation and mortality compared with conventional oxygen therapy (COT).^1^ However, studies prior to the COVID-19 pandemic comparing NIV (including CPAP) and HFNO include patients with heterogeneous underlying aetiologies of ARF and illness severity, which might confound direct comparisons between NIV and HFNO, and COT. Additionally, previous studies investigating the use of noninvasive respiratory support included studies dating as early as 1995, when the management and outcomes of acute respiratory distress syndrome (ARDS) were different.^2^

The COVID-19 pandemic provided an opportunity to understand the utility of noninvasive respiratory support to prevent patients progressing to requiring IMV or mortality, among a homogeneous cohort of patients with present-day management of ARDS. We therefore performed a network meta-analysis of studies evaluating the efficacy of NIV (including CPAP) and HFNO, compared with COT, in preventing patients with COVID-19 requiring IMV.

## Methods

This review follows a protocol that was registered with the International Prospective Register of Systematic Reviews (PROSPERO registration number: CRD42023426495) and is reported in accordance with PRISMA guidelines.

### Information sources and search strategies

A search was conducted on PubMed, Embase, and Cochrane Library, using MeSH terms and keywords without language restrictions. A date restriction was applied to include only papers published from 2019 onwards. The search was last updated on May 12, 2023. The Boolean search strategy used was: (ARDS OR acute respiratory distress syndrome OR COVID-19 OR SARS-CoV-2) AND (respiratory support OR positive end expiratory pressure OR high flow oxygen OR optiflow OR HFNO OR CPAP OR helmet OR facemask OR conventional oxygen OR venturi OR ventilation) AND (randomised OR randomized OR clinical trial) NOT (animal OR neonate OR pediatrics OR paediatrics OR Children). Additionally, relevant research papers and review articles were manually searched. In cases where primary outcome data were not present in the manuscript, the corresponding author was contacted for the required information.

### Eligibility criteria

According to the PICOS framework (Centre for reviews and dissemination. *Systematic Reviews: CRD's Guidance for Undertaking Reviews in Health Care.* York: University of York; 2006),^3^ the study's inclusion criteria were established as follows: (1) only RCTs were considered, (2) which enrolled adults with COVID-19-related ARF, (3) and compared NIV with COT, HFNO with COT, or NIV with HFNO, (4) with reported outcomes of progression on IMV, 20- to 30-day mortality at hospital discharge, or both. Trials involving paediatric patients (<18 yr), studies published as a meeting abstract, or non-randomised studies were excluded.

### Trial selection

Two researchers (WP and PA) independently screened both titles and abstracts of identified articles and selected the relevant ones. Discrepancies were resolved by a third author (AP). Relevant full-text articles were retrieved and analysed for eligibility.

### Data collection and analysis

The data extracted included first author, study group, country of trial, recruitment dates, timing of HFNO/NIV/COT initiation, sample size, patient characteristics, *P*a_o2_:FiO_2_ ratio at enrolment, concomitant therapies for COVID-19, progression to IMV, and mortality.

### Primary and secondary outcomes

Primary outcome was treatment failure, defined as discontinuation of HFNO, NIV, or COT despite progressive disease. Secondary outcome was mortality (either 30-day, 90-day, or in-hospital as reported in the individual studies).

### Risk of bias assessment

Two authors (KA and WP) independently assessed the methodological quality of the RCTs included in the study using the Cochrane Collaboration's risk of bias tool (RoB 2).^4^ Any discrepancies were resolved by a third author (NA). The authors evaluated the following domains: randomisation process, assignment to intervention, missing outcome data, measurement of outcome, selection of the reported result, other bias, and overall bias. Each domain was evaluated as having either low risk of bias, high risk of bias, or some concerns.

### Statistical analysis

Standard random-effects meta-analysis (using restricted maximum likelihood, REML) was used first to estimate all direct pairwise associations between the treatments and the outcome. Then, the results from all studies were combined using frequentist network meta-analysis.^5^ This was performed in Stata using multivariate meta-analysis and meta-regression methodology.^6^ We fitted consistency models and used these to estimate the probability that each treatment being the best. Then, where possible, we fitted inconsistency models and performed tests of inconsistency. The results are presented using forest plots and rankograms, where rankograms display the cumulative probability of each treatment having the greatest effect size. The rankograms present cumulative probability, so only the ‘best’ percentage sums to 100%.

One study investigated all three treatments and presented the results as pairwise comparisons (see later section).^7^ However, not all patients were randomised to three treatments; therefore, in our main network analysis, we just included the results from their NIV and HFNO comparison. In sensitivity analyses, we include the results from the other comparisons.

## Results

### Search strategy

**Fig 1:**
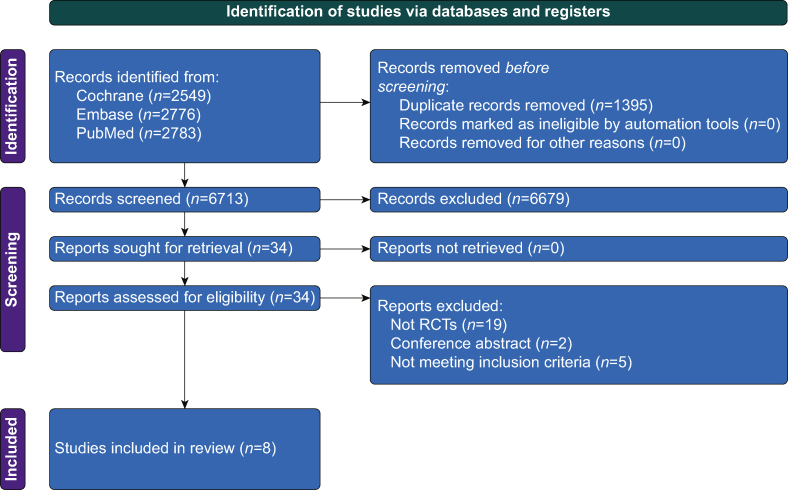
PRISMA flow chart for the systematic review.

Our search strategy identified 8108 results (Fig. 1). After removal of duplicates, 6713 articles remained. Of these, 6679 were excluded on the basis of title/abstract. Of the remaining 34 studies, 26 studies were excluded after full review, leaving eight studies. Among the studies excluded after full-text review, 19 were not RCTs,^8^, ^9^, ^10^, ^11^, ^12^, ^13^, ^14^, ^15^, ^16^, ^17^, ^18^, ^19^, ^20^, ^21^, ^22^, ^23^, ^24^, ^25^, ^26^ two were conference abstracts,^27^^,^^28^ and five did not meet inclusion criteria.^29^, ^30^, ^31^, ^32^, ^33^ The Helmet-COVID RCT, comparing helmet-NIV *vs* usual respiratory support did not meet inclusion criteria because it included facemask-NIV as part of usual respiratory support.^29^

### Study characteristics

**Fig 2:**
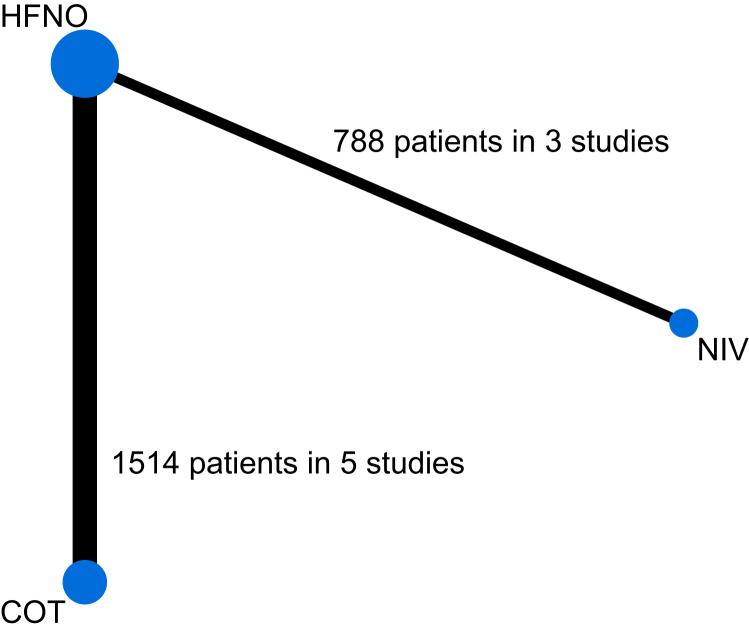
Illustration of which treatments are compared against which other treatments, with the width of the line reflecting the amount of information available for each comparison. COT, conventional oxygen therapy; HFNO, high-flow nasal oxygen; NIV, noninvasive ventilation.

We included data from eight studies in the network meta-analysis (Table 1). Among the 2302 patients, 756 (33%) were assigned to the COT group, 371 (16%) to the NIV group, and 1175 (51%) to the HFNO group (Fig. 2). Five studies compared HFNO with COT,^34^, ^35^, ^36^, ^37^, ^38^ two studies compared HFNO with NIV,^39^^,^^40^ and one study investigated all three treatments and presented the results of all pairwise comparisons across three tables (albeit most patients were randomised to two of the three treatments).^7^ However, to avoid double-counting patients, we only include the results from their NIV *vs* HFNO comparison in our main network analysis (Supplementary Table S1). The results of other comparisons are included in the pairwise meta-analyses and sensitivity analyses.

**Table 1:** Demographics and patient characteristics of included studies. COT, conventional oxygen therapy; HFNO, high-flow nasal oxygen; NIV, noninvasive ventilation.

Author/group/NCT registration	Country	Total number of patients	Main exposure	Com parator	Age (mean range), yr	Age NIV group, yr	Age HFNO group, yr	Age COT group, yr	Sex (% male)	Sex (% male) NIV group	Sex (% male) HFNO group	Sex (% male) COT group	*P*a_o2_:FiO2 ratio mean (sd)	*P*a_o2_:FiO2 ratio mean (sd) NIV group	*P*a_o2_:FiO2 ratio mean (sd) HFNO group	*P*a_o2_:FiO2 ratio mean (sd) COT group	Timing of measurement for study treatment failure and mortality
Frat JP/SOHO-COVID/NCT04468126*JAMA* 2022^37^	France	711	HFNO (*n*=357)	COT (*n*=354)	61 (12)	–	61 (12)	61 (12)	70	–	70	70	130 (31)	–	128 (31)	132 (31)	28-day, 28-day
Grieco DL/HENIVOT/NCT04502576*JAMA* 2021^39^	Italy	109	NIV (*n*=54)	HFNO (*n*=55)	63 (11)	64 (11)	62 (10)	–	81	77	84	–	103 (32)	104 (31)	102 (33)	–	28-day, 28-day
Ospina-Tascón GA/HiFlo-COVID/NCT04609462*JAMA* 2021^36^	Colombia	199	HFNO (*n*=99)	COT (*n*=100)	59 (15)	–	59 (14)	59 (15)	67	–	72	63	114 (42)	–	111 (37)	116 (47)	28-day, 28-day
Perkins GD/RECOVERY-RS/ISRCTN16912075*JAMA* 2022^7^	UK and Jersey	1273	CPAP (*n*=380) or HFNO (*n*=418)	COT (*n*=475)	57 (13)	57 (13)	58 (13)	58 (13)	66	68.4	65.1	65.7	130 (70)	130 (73)	135 (80)	124 (55)	30-day, 30.day
Crimi C/COVID-HIGH/NCT04655638*Thorax* 2023^34^	Italy, Greece, Spain, Portugal, Poland, Turkey	362	HFNO (*n*=181)	COT (*n*=181)	59 (15)	–	59 (15)	59 (15)	64	–	65.7	61.9	274 (22)	–	272 (22)	276 (21)	28-day, 28-day
Nazir *Thoracic Crit Care Med* 2022^35^	India	120	HFNO (*n*=60)	COT (*n*=60)	55 (9)	–	54 (12)	57 (3)	50	–	47	53	208 (5)	–	207 (5)	208 (4)	28-day, 28-day
Nair PR/CTRI/2020/07/026835 (Clinical Trials Registry of India)*Respir Care* 2021^40^	India	109	NIV (*n*=54)	HFNO (*n*=55)	56 (12)	56 (12)	56 (12)	–	72	64.8	80	–	119 (42)	121 (45)	116 (38)	–	7-day, in-hospital mortality
Thota B/CTRI/2020/12/029803 (Clinical trial registry, India)*Indian J Anaesth* 2022^38^	India	122	HFNO (*n*=61)	COT (*n*=61)	59 (12)	–	60 (10.3)	58 (12.6)	70	–	72	67	SpO_2_/FiO_2_: 243 (54)	–	SpO_2_/FiO_2_: 235 (55)	SpO_2_/FiO_2_: 250 (53)	ICU discharge, 28-day

Studies were conducted between April 2020 and December 2021. Study size ranged from 109 to 1273 patients. The mean age of patients was 59 (±13) in the COT group, 59 (±13) in the HFNO group, and 58 (±13) in the NIV group. The *P*aO_2_:FiO_2_ (in mm Hg) ratio on study enrolment was reported in all but one study,^38^ which reported the SpO_2_:FiO_2_ ratio. The mean *P*aO_2_:FiO_2_ ratio of patients was 164 (sd 73) in the COT group, 159 (75) in the HFNO group, and 164 (73) in the NIV group.

### Primary outcome: treatment failure

Treatment failure was reported in all included studies, although the definition and evaluation time points varied across them. Specifically, five studies^7^, ^36^, ^37^, ^39^, ^40^ assessed the proportion of patients requiring endotracheal intubation, with one study^40^ evaluating it at 7 days from admission and the other four studies at 28–30 days from enrolment or randomisation.^7^^,^^36^^,^^37^^,^^39^ Three studies^34^^,^^35^^,^^38^ defined treatment failure as the escalation of respiratory support to a different device or modality.

**Fig 3:**
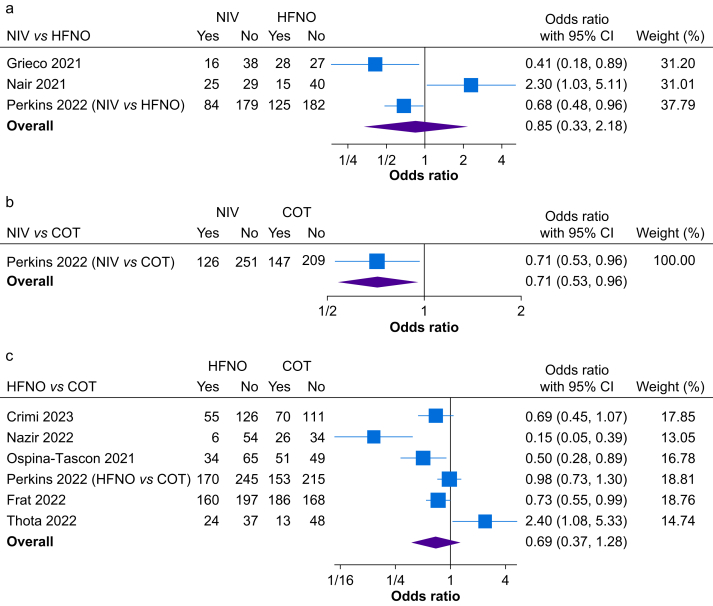
Forest plots of pairwise comparisons between (a) NIV/CPAP (Treatment) *vs* HFNO (Control), (b) NIV/CPAP (Treatment) *vs* COT (Control), and (c) HFNO (Treatment) *vs* COT (Control) for treatment failure. COT, conventional oxygen therapy; CPAP, continuous positive airway pressure; HFNO, high-flow nasal oxygen; NIV, noninvasive ventilation.

The results from the pairwise meta-analyses are shown in Fig. 3. There is considerable heterogeneity across the studies. Three studies compared NIV with HFNO, and the pooled result suggests similar rates of treatment failure between groups (odds ratio [OR]=0.85 [0.32–2.18]; *P*=0.73).^7^, ^39^, ^40^ One study compared CPAP with COT, which demonstrated that CPAP was associated with lower incidence of treatment failure compared with COT (OR=0.71 [0.53–0.96]; *P*=0.03).^7^ Six studies compared HFNO with COT, and there was no significant difference in the rates of treatment failure between groups (OR=0.69 [0.37–1.28]; *P*=0.24).^7^^,^^34^, ^35^, ^36^, ^37^, ^38^

**Fig 4:**
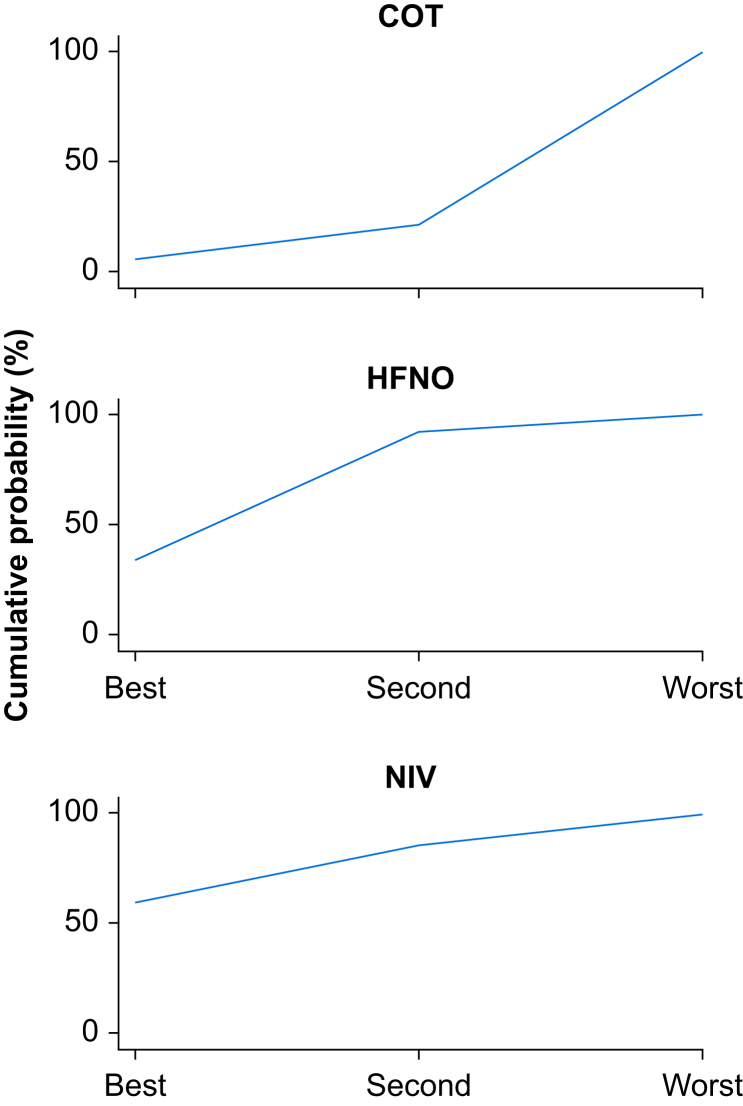
Rankogram showing the cumulative probability of each treatment being ranked best, second, or worst. COT, conventional oxygen therapy; HFNO, high-flow nasal oxygen; NIV, noninvasive ventilation.

We performed network meta-analysis. The results from the consistency model (Table 2) suggest that the odds of treatment failure are similar for NIV and HFNO, and both are lower than that for COT (the reference category), although neither comparison is significant at the 0.05 level (*P*=0.33 and 0.25, respectively). The rankogram shows the cumulative probability that each treatment is best. The estimated probability of NIV being best is 60.2% compared with 34.3% for HFNO (Fig. 4). However, neither comparison was statistically significant. We were unable to fit an inconsistency model for this analysis as there was no direct NIV *vs* COT comparison.

**Table 2:** Results from the consistency model. COT, conventional oxygen therapy; HFNO, high-flow nasal oxygen; NIV, noninvasive ventilation.

Comparison	Odds ratio (95% confidence interval)	*P*-value
NIV *vs* COT	0.539 (0.154–1.881)	0.333
HFNO *vs* COT	0.636 (0.296–1.365)	0.246

We repeated the network meta-analysis, including different data from the study by Perkins and colleagues.^7^ When including just the NIV *vs* COT comparison, the estimated probability of NIV being best was 47.6% compared with 47.5% for HFNO; which were both higher than that for COT. When we included just the HFNO *vs* COT comparison, the estimated probability of NIV being best was 49.5% compared with 42.4% for HFNO. Results from all comparisons suggest that HFNO and NIV are superior to COT in preventing treatment failure.

### Secondary outcome: mortality

Mortality was reported in all included studies. Six studies assessed mortality at 28-day,^34^, ^35^, ^36^, ^37^, ^38^, ^39^ one at 30-day,^7^ and one in-hospital mortality.^40^ The results from the pairwise meta-analyses are shown in Supplementary Figure S1. There is a moderate amount of heterogeneity across the studies. Three studies compared HFNO with NIV, and the results suggest that mortality is similar between the groups (OR=1.089 [0.60–1.99]; *P*=0.78).^7^, ^39^, ^40^ One study compared NIV with COT, and there was no statistical evidence to suggest that NIV was associated with lower mortality compared with COT (OR=0.84 [0.58–1.23]; *P*=0.37). Six studies compared HFNO with COT, which demonstrated very similar rates of mortality between groups (0.97 [0.68–1.38]; *P*=0.85).^7^, ^34^, ^35^, ^36^, ^37^, ^38^

We performed a network meta-analysis. The results from the consistency model (Table 3) suggest that the odds of mortality are similar for all three treatments (NIV *vs* COT: OR=1.06 [0.46–2.44] and HFNO *vs* COT: OR=0.97 [0.57–1.65]). The rankogram suggests that all three treatments have a similar probability of being best: COT (34.7%), HFNO (33.7%), and NIV (31.6%) (Supplementary Fig. S2).

**Table 3:** Results from the consistency model (mortality). COT, conventional oxygen therapy; HFNO, high-flow nasal oxygen; NIV, noninvasive ventilation.

Comparison	Odds ratio (95% confidence interval)	*P*-value
NIV *vs* COT	1.065 (0.464–2.443)	0.882
HFNO *vs* COT	0.973 (0.573–1.652)	0.919

We repeated the network meta-analysis, including different data from the study by Perkins and colleagues.^7^ When including just the NIV *vs* COT comparison, HFNO had the highest estimated probability of being best (46.9%), followed by NIV (29.3%) and COT (23.8%). When we included just the HFNO *vs* COT comparison, HFNO still had the highest estimated probability of being best (45.6%), this time followed by COT (35.7%) and NIV (18.7%).

### Risk of bias analysis

The risk of bias assessment revealed that two studies raised some concerns^7^^,^^40^ (Table 4). The remaining six studies were considered to have a low risk of bias. Information on rationale behind the risk of bias judgements are detailed in Supplementary Table S2.

**Table 4:**
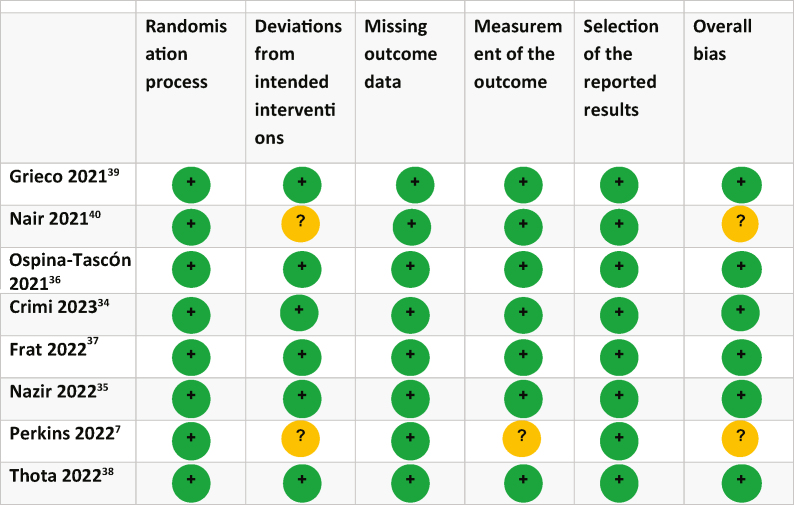
Risk of bias analyses.

## Discussion

We conducted a systematic review and network meta-analysis aimed to evaluate the efficacy of HFNO, COT, and NIV on treatment failure and mortality in a homogeneous cohort of patients with COVID-19-associated ARF. Our data suggest no difference in treatment failure and mortality associated with either NIV or HFNO compared with COT in COVID-19. In contrast, among patients with non-COVID-19 acute hypoxaemic respiratory failure, NIV and HFNO were associated with reduced risk of tracheal intubation, and NIV associated with lower risk of mortality than COT.^1^

A strength of our analyses compared with previous meta-analyses includes the lack of heterogeneity in ARDS aetiologies, allowing for a better comparison between respiratory support modalities. Additionally, all the studies included in this review were conducted during the COVID-19 pandemic, which may be associated with a more consistent approach to management of patients with acute lung injury and ARDS. These interventions, along with improvements in overall supportive care, have led to better outcomes in ARDS over time.^2^

However, it may not be possible to extrapolate findings in COVID-19 ARDS to non-COVID ARDS as patients with non-COVID-19 ARDS may not respond to positive pressure ventilation in the same way as patients with COVID-19. Early in the COVID-19 disease process, hypoxemia develops despite good pulmonary compliance, and a pulmonary vasculopathy is implicated.^41^ A limited improvement in oxygenation to inhaled nitric oxide treatment supports the latter phenomenon.^42^ A decrease in pulmonary compliance to that seen with ‘classical’ ARDS may develop later in the disease.^41^^,^^43^

Our analysis focused on the efficacy of respiratory support modalities and their impact on treatment failure and mortality. The optimal timing of initiation of IMV was not evaluated in this study. Early initiation of IMV has been suggested to prevent the development of severe lung injury secondary to self-induced lung injury.^44^^,^^45^

Limitations of our study should also be acknowledged. Despite limiting our study to a single ARDS aetiology, there was heterogeneity among the included studies in the severity of ARDS based (e.g. mean P/F ratio on study inclusion). Additionally, there were variations in the use of adjunctive therapies for COVID-19 among between studies. We were not able to factor in the effect of the different timing and setting during the course of the pandemic on use of noninvasive respiratory support. We have grouped CPAP and NIV as a single treatment modality, as there are insufficient data (with two NIV and one CPAP studies) to assess the two modalities independently.

NIV can be delivered via different interfaces such as helmets and facemasks, which may influence the tolerability and thus success of NIV use.^46^ We were not able to account for the differences in baseline hypoxaemia, adjunctive COVID-19 treatments, nor the different modalities of NIV interfaces used between different studies. Additionally, the limited number of studies/patients available may impact the robustness of our findings.

Future perspectives should focus on conducting RCTs with larger sample sizes to provide more robust evidence on the comparative effectiveness of NIV, HFNO, and COT in the management of ARDS. Additionally, studies evaluating the optimal timing of IMV and the potential benefits of combining different respiratory support modalities are warranted.

The results of our network meta-analysis suggest that noninvasive ventilation, high-flow nasal oxygen, and conventional oxygen therapy are comparable in terms of treatment failure and mortality outcomes in COVID-19-associated acute respiratory failure. Although there is a trend towards improved outcomes with NIV and high-flow nasal oxygen compared with COT, the evidence remains inconclusive. High-flow nasal oxygen consistently ranked best with respect to mortality in sensitivity analyses, but further research is needed to strengthen the evidence base and provide more definitive conclusions.

## Authors’ contributions

Study design: NA

Data collection: WP, AP, PA, KA

Statistics: GA

Drafting manuscript: WP

Finalising manuscript: GA, NA

## Declaration of interest

The authors declare that they have no conflicts of interest.

## Funding

Salary funding from Medical research council (MRC) (MR/W030489/1 to NA).
