# Supplementary material for: High-flow nasal oxygen versus conventional oxygen therapy and noninvasive ventilation in COVID-19 respiratory failure: a systematic review and network meta-analysis of randomised controlled trials
Source: Br J Anaesth. 2024 Feb 2;132(5):936–44. doi: 10.1016/j.bja.2023.12.022 (PMC11103093; doi:10.1016/j.bja.2023.12.022)
Supplement: Multimedia component 1 [file mmc1.docx]

**Supplementary data**

|  |  | COT | | NIV | | HFNO | |
| --- | --- | --- | --- | --- | --- | --- | --- |
|  | Study | Events | Total number of patients | Events | Total number of patients | Events | Total number of patients |
| 1 | Crimi 2021 | 70 | 181 |  |  | 55 | 181 |
| 2 | Grieco 2021 |  |  | 16 | 54 | 28 | 55 |
| 3 | Nair 2021 |  |  | 25 | 54 | 15 | 55 |
| 4 | Nazir 2022 | 26 | 60 |  |  | 6 | 60 |
| 5 | Ospina 2021 | 51 | 100 |  |  | 34 | 99 |
| 6 | Frat 2022 | 186 | 354 |  |  | 160 | 357 |
| 7 | Thota 2022 | 13 | 61 |  |  | 24 | 61 |
| 8 | Perkins 2022 (1) |  |  | 84 | 263 | 125 | 307 |
| 8 | Perkins 2022 (2) | 147 | 356 | 126 | 377 |  |  |
| 8 | Perkins 2022 (3) | 153 | 368 |  |  | 170 | 415 |

**Supplementary Table 1**: Outcome data for treatment failure for each study. Perkins (1) only was used in the network meta-analysis whereas Perkins (1)-(3) were used in the pairwise meta-analyses and sensitivity analyses.

| Author, year | Randomisation process | Deviations from intended interventions | Missing outcome data | Measurement of the outcome | Selection of the reported results |
| --- | --- | --- | --- | --- | --- |
| Grieco 2021 | - computer-generated | - Patients, clinicians were not blinded | - Reported data of all enrolled patients | - Outcome assessors were blinded | - Low risk |
| Nair 2021 | - computer-generated | - Patients, clinicians were not blinded - It’s unclear if there was crossover between groups | - Reported data of all enrolled patients | - Outcome assessors were blinded | - Low risk |
| Ospina 2021 | - computer-generated | - Patients, clinicians were not blinded - It seems that does not influence the clinical outcomes | - Reported data of all enrolled patients | - Outcome assessors were blinded | - Low risk |
| Crimi 2022 | - computer-generated | - Patients, clinicians were not blinded - More protocol violations in control group vs interventional (26 vs 2), not balanced; however they were not due to trial context, and they were appropriately analysed (Intention to treat analysis) | - Reported data of all enrolled patients | - Outcome assessors were un-blinded - Unclear if this led to an influence on the assessor’s judgment | - Low risk |
| Frat 2022 | - computer-generated | - Patients, clinicians were not blinded - However, that’s probably did not affect the intended intervention | - Reported data of all enrolled patients | - Outcome assessors were blinded | - Low risk |
| Nazir 2022 | - computer-generated | - Patients, clinicians were not blinded - No protocol deviations | - Reported data of all enrolled patients | - Blinding to outcome assessors were not explained - Unclear if this led to an influence on the assessor’s judgment, but it’s unlikely | - Low risk |
| Perkins 2022 | - computer-generated | - Patients, clinicians were not blinded - Cross-over between groups are not balanced | - Some missing data - It seems the reason of the missing data is not its true value | - Outcome assessors were unblinded - Unclear if this led to an influence on the assessor’s judgment, but it’s unlikely | - Low risk |
| Thota 2022 | - computer-generated | - Patients, clinicians were not blinded - All patient received allocated intervention and no protocol violation reported | - Reported data of all enrolled patients | - Blinding of outcome assessors were not mentioned; however, we think unblinding will not affect the results as the measured outcomes were 28-day mortality and escalation to advanced treatment | - Low risk |

**Supplementary Table 2**: Risk of bias analysis grading rationale.


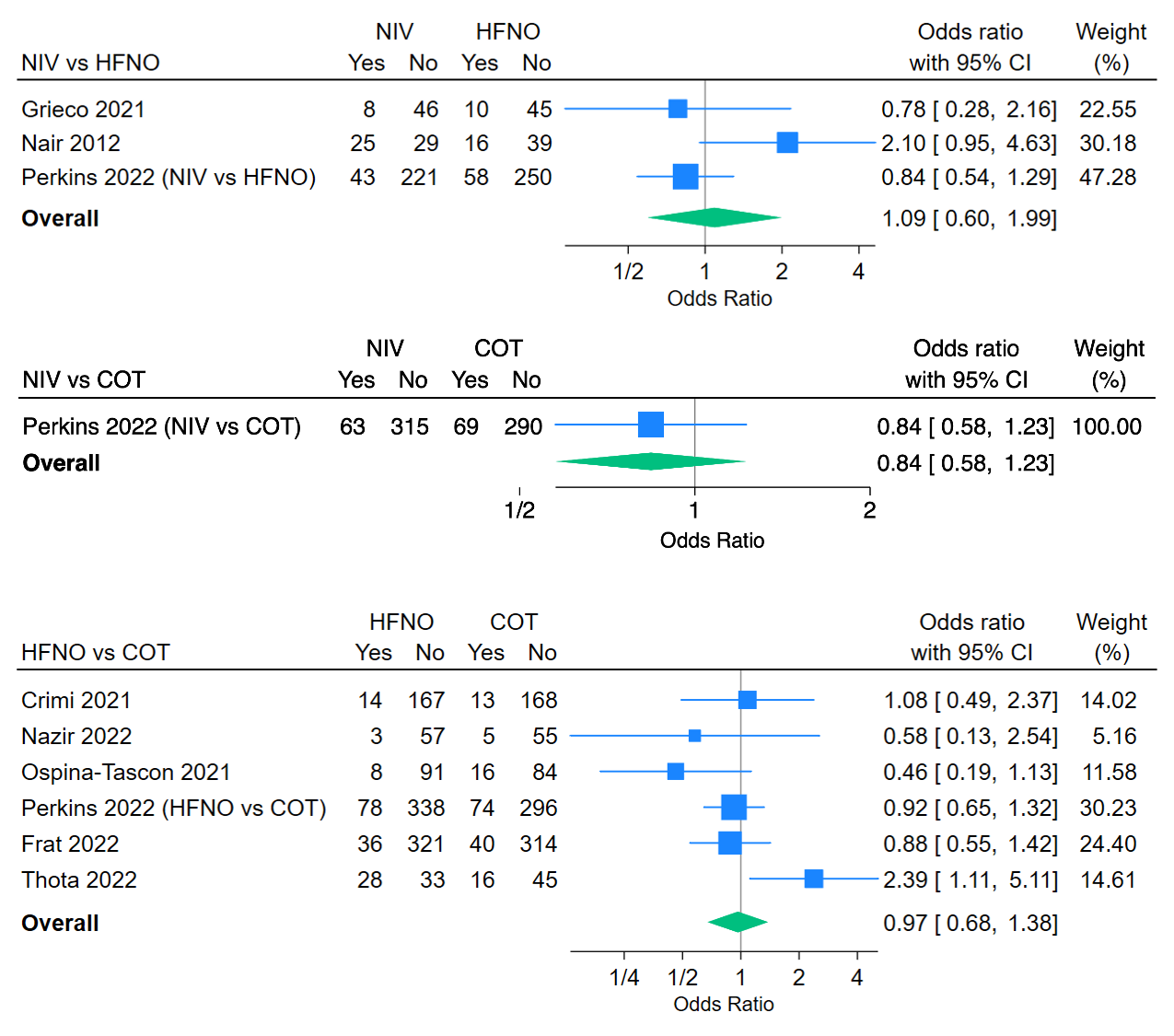


**Supplementary Figure 1:** Forest plots of pairwise comparisons between (a).NIV (Treatment) vs HFNO (Control) (b) NIV (Treatment) vs COT (Control), and (c) HFNO (Treatment) vs COT (Control) for mortality.

**
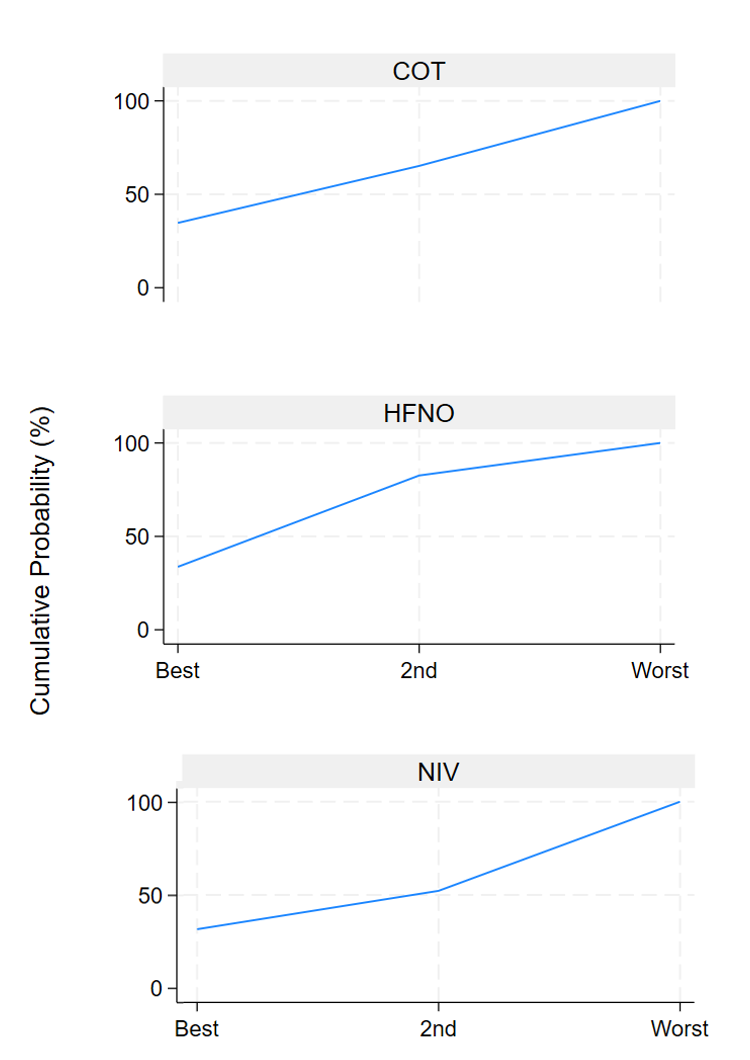
**

**Supplementary Figure 2:** Rankogram showing the cumulative probability of each treatment being ranked best, 2^nd^ or worst in relation to mortality.
